# Latest advances on new promising molecular-based therapeutic approaches for Huntington’s disease

**DOI:** 10.2478/jtim-2023-0142

**Published:** 2024-05-21

**Authors:** Yangfan Cheng, Sirui Zhang, Huifang Shang

**Affiliations:** Department of Neurology, Laboratory of Neurodegenerative Disorders, Rare disease center, West China Hospital, Sichuan University, Chengdu 610041, Sichuan Province, China

**Keywords:** Huntington’s disease, preclinical and clinical studies, therapeutic strategy

## Abstract

Huntington’s disease (HD) is a devastating, autosomal-dominant inherited, neurodegenerative disorder characterized by progressive motor deficits, cognitive impairments, and neuropsychiatric symptoms. It is caused by excessive cytosine-adenine-guanine (CAG) trinucleotide repeats within the huntingtin gene (HTT). Presently, therapeutic interventions capable of altering the trajectory of HD are lacking, while medications for abnormal movement and psychiatric symptoms are limited. Numerous pre-clinical and clinical studies have been conducted and are currently underway to test the efficacy of therapeutic approaches targeting some of these mechanisms with varying degrees of success. In this review, we update the latest advances on new promising molecular-based therapeutic strategies for this disorder, including DNA-targeting techniques such as zinc-finger proteins, transcription activator-like effector nucleases, and CRISPR/Cas9; post-transcriptional huntingtin-lowering approaches such as RNAi, antisense oligonucleotides, and small-molecule splicing modulators; and novel methods to clear the mHTT protein, such as proteolysis-targeting chimeras. We mainly focus on the ongoing clinical trials and the latest pre-clinical studies to explore the progress of emerging potential HD therapeutics.

## Introduction

Huntington’s disease (HD) is a devastating, autosomal-dominant inherited, neurodegenerative disorder characterized by progressive motor deficits, cognitive impairments, and neuropsychiatric symptoms.^[1]^ It is caused by a cytosine-adenine-guanine (CAG) repeat expansion in the first exon of the *Huntingtin* gene, located in the short arm of chromosome 4 (4p16.3),^[2]^ encoding for a mutant huntingtin (mHTT) protein. Worldwide, the latest meta-analysis of 20 studies published from 1985 to 2010 shows that the incidence and prevalence of HD is estimated at 0.38 per 100, 000 person-years (95% confidence interval [CI], 0.16–0.94) and 2.71 per 100, 000 persons (95% CI, 1.55–4.72), respectively,[^3^] while this varies regionally. HD tends to be more prevalent in populations of European descent, particularly in Western Europe and North America, while Asian countries tend to have a lower prevalence of the disease.^[4,5,6]^ The clinical manifestations of HD include involuntary movements (chorea), cognitive decline, psychiatric disturbances (such as depression, irritability and anxiety), and progressive functional impairment.^[7]^ The onset of these symptoms is typically insidious and follows a progressive course. HD gene carriers could remain unnoticed for several years before receiving a diagnosis.^[8]^ Disease manifestations could begin at any time in life, although the majority of cases display evident onset in middle age. The length of the CAG repeats accounts for about 50%–70% of the overall variance in age of onset.^[9,10]^ Higher numbers of CAG repeats are thought to be associated with an earlier onset of disease, a faster rate of clinical progression, and increasing disease severity.^[11]^ Present studies show that disrupted neural circuit physiology could be found in HD mice during the first neonatal week^[12]^ and cognitive impairments could happen earlier than the motor symptoms even in the second decades of patients,^[13,14]^ which suggests the disease onset is the cumulative effect and the need for disease-modifying drugs of HD is urgent. Diagnosis of HD is typically based on clinical features, family history, and genetic testing to confirm the presence of the CAG repeat expansion in the HTT gene.^[1]^ Death occurs at a median of 18 years after symptom onset, with infections, specifically aspiration pneumonia, being the most common cause of death.^[15,16]^ Currently, there is no cure for HD, and available treatments focus on managing symptoms and improving the quality of life for affected individuals. Supportive therapies, including physical and occupational therapy, speech therapy, and medications to alleviate motor and psychiatric symptoms, form the cornerstone of management.^[17]^

The molecular pathogenesis of HD is complex,^[18]^ with toxicity that arises from full-length expanded and N-terminal fragments of HTT protein, which are both prone to misfolding due to proteolysis;^[19,20,21]^ aberrant intron-1 splicing of the HTT gene;^[22,23]^ and somatic expansion of the CAG repeat in the HTT gene.^[24]^ Therefore, it is thought to arise predominantly from a toxic gain-of-function of the mHTT protein.^[25,26]^ The pathological mechanisms include early transcriptional dysregulation, synaptic dysfunction, altered axonal trafficking, impairment of the proteostasis network, aggregate pathology, impairment of the nuclear pore complex function, oxidative damage, mitochondrial dysfunction, and extra-synaptic excitotoxicity,^[20,27,28]^ inducing the progressive degeneration of gamma-aminobutyric acid (GABA)-ergic medium spiny neurons (MSNs) initially within the striatum.^[29]^ Then degeneration subsequently extends to the broader brain cortex, affecting regions responsible for motor control and cognition.^[30]^ Currently, the focus on potential interventions for HD includes therapies targeting HTT DNA and RNA, clearance of HTT protein, and DNA repair pathways. These approaches ultimately aim to reduce mHTT levels and, therefore, ameliorate all of its downstream pathogenic effects, which are multiple and varied. In this review, we provide an update on the latest advances in molecular-based therapeutic approaches targeting the HTT mutation and toxic species relevant to HD pathogenesis.

## Promising molecular-based therapeutical approaches

**Figure 1:**
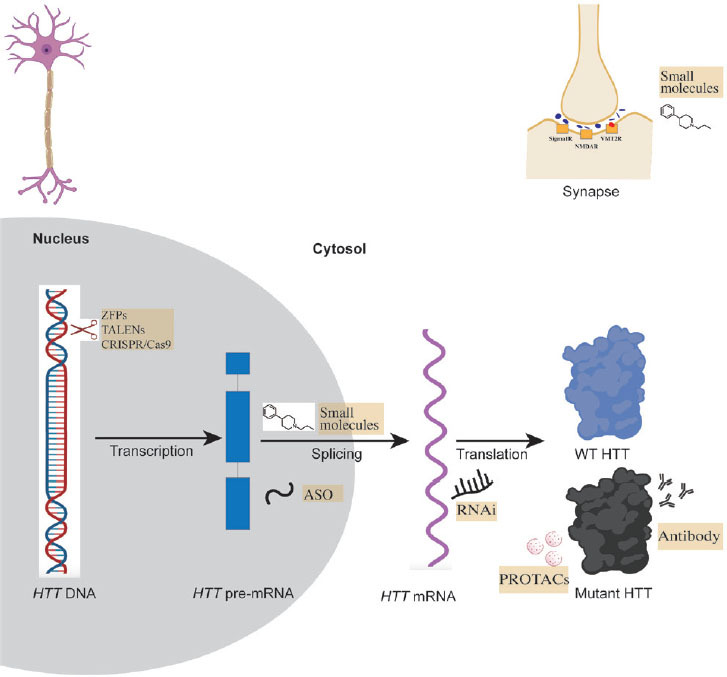
Molecular-based therapeutical approaches for HD. The orange boxes are therapeutic approaches. ASO: antisense oligonucleotide; CRISPR: clustered regularly interspaced short palindromic repeats; mHTT: mutant huntingtin; NMDAR: N-methyl-d-aspartate receptor; PROTACs: proteolysistargeting chimeras; RNAi: RNA interference; Sigma1R: sigma1 receptor; TALENs: transcription activator-like effector nucleases; VMT2R: vesicular monoamine transporter 2; WT-HTT: wildtype huntingtin; ZFP: zinc-finger protein.

Recent and ongoing clinical trials are exploring a range of treatments for HD, from symptomatic management to interventions that modify the disease’s underlying pathogenesis. There has been a notable increase in the number of HD clinical trials over time, with a significant rise in participant numbers. These trials are diverse, encompassing various approaches such as RNA/DNA targeting therapies, protein therapies, antibody therapies, and small molecules.^[16]^ In this review, we are going to introduce new and promising molecular-based therapeutic approaches targeting HD, illustrated in Figure 1. We have also compiled the most promising molecular-based clinical trials in Table 1 and preclinical trials in Table 2, categorizing them based on their therapeutic targets.

**Table 1:** The current promising molecular-based clinical trials for HD based on different therapeutical targets.

Intervention	mechanisms	Name	CT Identifier	Allocation	Phase	Stage	Enrollment (participants)
ASO therapy (Pre-mRNA degradation)		Tominersen (RG6042)	NCT03761849 (GENERATION HD1)	Randomized	3	Completed;	791
	Allele-nonselective ASO	Tominersen (RG6042)	NCT05686551 (GENERATION HD2)	Randomized	2	Recruiting	360
		Tominersen (RG6042)	NCT03842969 (GEN-EXTEND)	Randomized	3	Completed;	1050
	Allele-selective ASO	WVE-120101	NCT03225833 (PRECISION-HD1)	Randomized	1b/2a	Terminated (Lack of Efficacy	61
		WVE-120102	NCT03225846 (PRECISION-HD2)	Randomized	1b/2a	Terminated (Lack of Efficacy	88
	CAG-expansion-specific ASO	WVE-003	NCT05032196 (SELECT-HD)	Randomized	1b/2a	Recruiting	36
		VO659	NCT05822908	Non-Randomized	1/2a	Recruiting	65
RNA interference	Non-selective HTT lowering gene therapy	AMT-130	NCT05243017	Non-Randomized	1b/2a	Recruiting	15
			NCT04120493	Randomized	1/2	Recruiting	44
	A selective agonist of the sigma1R	Pridopidine	NCT04556656	Randomized	3	Active, not recruiting	499
Reversibly inhibit the human VMAT2	A monoamine-depleting agent	Deutetrabenazine	NCT04713982	Single-arm study	2/3	Recruiting	30
			NCT04071639	Non-Randomized	1	Recruiting	60
			NCT04301726	Randomized	1	Not yet recruiting	48
Increase the production of BDNF	Derivative of the endogenous steroid 24 (S)-hydroxycholesterol	SAGE-718	NCT05107128	Randomized	2	Recruiting	178
positive allosteric modulator of the NMDA receptor	Positive allosteric modulator of the NMDA receptor	Branaplam	NCT05358821	Randomized	2	Recruiting	80
			NCT05655520	Non-Randomized	3	Recruiting	300
			NCT05111249 (VIBRANT-HD)	Randomized	2	Completed	75
Modulating HTT’s alternative splicing to lower huntingtin levels	mRNA splicing modulator	PTC 518	NCT05358717	Randomized	2a	Recruiting	252
lower huntingtin levels	Selective VMT2	Valbenazine	NCT04400331	N/A	3	Active, not recruiting	154
Symptomatic study: chorea	Alternative VMAT2 inhibitor	SOM3355 (Bevantolol hydrochloride)	NCT05475483	Randomized	2	Recruiting	129
Symptomatic study: chorea	NMDA receptor antagonist and sigma 1 agonist	Dextromethorphan/ quinidine	NCT03854019	Randomized	3	Completed (at May 16, 2023)	20
Symptomatic study: Irritability	/	Melatonin	NCT04421339	Randomized	Not Applicable	Completed (at May 15, 2023)	15
Symptomatic study: circadian rhythm sleep disorders	/	Oral NAC	NCT05509153	Randomized	2	Active, not recruiting	160
/	PPAR-α agonist	Fenofibrate	NCT03515213	Randomized	2	Completed	20
Symptomatic study: Cognitive decline	/	Metformin	NCT04826692	Randomized	3	Recruiting	60

HD: Huntington’s disease; ASO: antisense oligonucleotide; BDNF: Brain-derived neurotrophic factor; NAC: N-Acetyl Cysteine; NMDA: N-methyl-d-aspartate; Sigma1R: sigma1 receptor; VMT2: vesicular monoamine transporter 2.

**Table 2:** The current promising molecular-based preclinical trials for HD based on different therapeutical targets

Target	class	mechanisms	Name	Allele selectivity	Sponsor	Disease model
RNA	RNA interference	mRNA degradation	conjugation of 2’-O-palmityl (C16) to siRNAs	Nonselective	Alnylam Pharmaceuticals	Rat model
DNA	polyCAG-targeting ZFTR	Transcriptional repression	/	CAG repeat	Shire	R6/2 mice
	CRISPR/Cas9	Genome editing	/	SNP-targeted/ Nonselective HTT depletion by polyQ domain deletion	Harvard University Emory University	R6/2 mice
Mutant HTT	PROTACs	leverage ubiquitin-proteasome system for protein degradation	Molecular Glues	Allele-selective	Arvinas Operations	primary fibroblasts from patients with HD; Drosophila; HD-knock-in mouse model (HdhQ7/ Q140)
DNA regions with long expanded CAG repeats	Small molecules	genetic interference	specific transcription elongation cofactors	expanded CAG repeats	Nuredis	HD patient lymphoblastoid cells/ R6/2 mouse model of HD
C1q protein	monoclonal antibody	inhibit C1q	ANX005	/	Annexon Bioscience	Sprague Dawley rats, cynomolgus monkeys
HTT protein	antibody	increases the amount of HTT cleanup being performed by microglia	ATLX-1095	/	Alchemab Therapeutics	in animal models
mutant HTT	antibody	selectively binds mutant HTT (but not normal HTT) with the goal of removing toxic mutant HTT and restoring neuronal health	VTX-003	/	VectorY company	HTT cell model

HD: Huntington’s disease; CRISPR: Clustered regularly interspaced short palindromic repeats; CSF: cerebrospinal fluid; PROTACs: proteolysis-targeting chimeras; ZFTRs: zinc finger transcription factors; CRISPR: Clustered regularly interspaced short palindromic repeats; CSF: cerebrospinal fluid; PROTACs: proteolysis-targeting chimeras; ZFTRs: zinc finger transcription factors.

### DNA targeting therapies

Three primary classes of nucleases can be engineered for DNA-targeting applications: zinc-finger proteins (ZFPs), transcription activator-like effector nucleases (TALENs), and Cas9 or other RNA-guided bacterial nucleases. These classes differ in their mechanisms of DNA binding and modes of action.^[31]^

ZFPs are custom-designed artificial nucleases that make double-strand breaks at specific sequences, enabling efficient targeted genetic modifications such as corrections, additions, gene knockouts and structural variations. ZFPs are composed of two domains:(i) a DNA-binding domain comprised of zinc finger modules, like zinc finger transcription factors (ZFTRs) to modulate gene expression. and (ii) the FokI nuclease domain that cleaves the DNA strand, like zinc finger nucleases (ZFNs).^[31,32]^ ZFPs could be designed theoretically to bind selectively to expanded CAG repeats versus normal CAG repeat lengths in HD, allowing relatively specific binding to the mutant HTT gene.^[33]^ The pre-clinical data shows that mHTT could be repressed by ZFP transcription factor.^[32]^ It is reported that ZFPs could lower mHTT expression in cell lines without significantly altering expression from the nonexpanded HTT allele or the expression of other nonexpanded CAG repeat-containing genes and reduce mHTT levels and improve some HD-like behavioral phenotypes in R6/2 mice delivered by using adeno-associated virus (AAV) vectors.^[34]^ These findings provide valuable support for the continued advancement of allele-specific ZFP repressors and ZFNs as potential therapeutic options for HD. One drawback of ZFP therapeutics is the generation of non-native proteins that can trigger inflammatory and immune responses, leading to neuronal damage and limited efficacy. ^[25]^To address this issue, Agustin-Pavon lab develops a polyCAG-targeting zinc finger transcription factor (ZFTR) that incorporated a non-viral promoter and a novel repressor element designed to be structurally similar to the host (mouse) protein, which could modify construct demonstrated more sustained silencing effects compared to other approaches using non-native protein sequences. Although the mHTT level gradually rebounded after over six months.^[35]^ How to optimize this limitation and administer a single treatment well in advance of symptom onset, aiming to prevent the development of the disease is the direction to further study.

TALENs contain a DNA binding domain linked to a nuclease effect domain, inducing double strand breaks and hence allowing for gene deletion or correction.^[36]^ TALENs are assumed theoretically to exhibit higher efficiency and specificity than ZFPs.^[20]^ However, all therapies of TALENs for HD were still in the preclinical or drug discovery stages. Investigations have not been performed in animal models nor patients. TALENs were firstly reported to successfully shorten CAG repeats in yeast cells models.^[37]^ Subsequently in HD patients-derived fibroblasts, TALENs were also reported to cause CAG-collapse in mutant HTT allele and significantly reduced mutant allele expression.^[38]^ Additionally, transcription activator-like effector with a transcriptional repressor was designed to target mutant allele-specific single nucleotide polymorphism (SNP) without nuclease activities and was also verified to decrease the expression of mutant HTT.^[38]^ These studies provide a foundation for DNA-targeting therapies of HD patients using TALE proteins.

Clustered regularly interspaced short palindromic repeats (CRISPR) and the accompanying CRISPR-associated system (Cas) together form the basis of a prokaryote immune system that recognizes and destroys foreign DNA. Cas9 nuclease can be combined with synthetic guide RNA (gRNA) to produce a construct that can cut DNA with high precision at any chosen site.^[39]^ CRISPR/Cas 9 is a rapidly evolving area of research in genetic diseases. There are a number of different potential mechanisms of action for HTT gene-directed editing using CRISPR/ Cas9-type approaches, including direct excision of CAG repeats to correct the mutation and generate two wild-type HTT alleles; targeted inactivation of the mutant allele, leading to a hemizygous null state;^[40,41]^ or non-allelespecific targeting of the HTT gene that would lower total HTT levels. In addition, it is possible that CRISPR/ Cas9-targeted epigenetic editing may also be an effective therapeutic approach for HD by altering HTT gene transcription without permanent genome modification.^[42]^ The preclinical studies showed that non-catalytic CRISPR/ Cas9 strategy could block HTT gene transcription in a human non-neuronal cell line.^[32]^ In HD patient fibroblasts, precise excision of the CAG repeats from the HTT gene has been accomplished using paired gRNA constructs that target DNA flanking the CAG repeat and a modified “nickase” Cas9 approach and results in decreased mHTT expression.^[32]^ Similarly, Cas9 is also successful in the Q140 transgenic mouse model of HD, producing selective mutant HTT reduction, attenuation of pathology, and improved motor function.^[43]^ Recently, the researchers in Jong-Min Lee lab develop an allele-specific CRISPR/Cas9 strategy to permanently inactivate mutant HTT through nonsense-mediated decay by capitalizing on exonic protospacer adjacent motif-altering (PAM-altering) SNP. The rs363099-based CRISPR/Cas9 shows perfect allele specificity and good targeting efficiencies in patient-derived cells, which could target to a predicted ~20% of HD subjects with European ancestry.^[41]^ CRISPR-based approaches are still in the early stages of development and are undergoing preclinical studies. However, these approaches face several challenges, including the potential for off-target effects, complexities in design and administration, and the activation of the neuroimmune response. These factors need to be carefully addressed and optimized before CRISPR-based therapies can progress to clinical trials and be considered as potential treatments for HD.^[44,45]^

### RNA targeting therapies

Starting from the antisense oligonucleotide (ASO) therapies, which is a promising molecular approach for treating a variety of genetic diseases by targeting and modulating specific genes or their transcripts.^[46]^ The concept of ASO therapy is based on the specific base pairing of a synthetic nucleotide sequence with the targeted mRNA, leading to the inhibition or degradation of the mRNA and thereby reducing the levels of the encoded protein.^[47]^ The causative mHTT gene of HD is the first logical therapeutic target. Suppressing the disease-causing mutation by targeting allele-selective lowering of mHTT is a potential treatment for HD.

Except for targeting the mutation selectively, a nonselective approach is also adopted to partially suppress both wild-type huntingtin (wtHTT) and mHTT. Although complete inactivation of the HTT is embryonically lethal, the fact that the HTT gene is haplosufficient indicates that deletion of one copy of HTT does not lead to an overt abnormal phenotype in the early life.^[48]^ Since the studies on HD models including cells, mice and nonhuman primates ^[49]^ have proved that non-selective ASO could decrease the expression of both mHTT and wtHTT protein without overting abnormal phenotype, suggesting that appropriately dosed nonselective ASOs could provide a potentially safe and effective therapy.^[50]^ The first study in humans has also confirmed the idea above^[51]^ (NCT02519036 and NCT03342053). For this reason, Roche started a global study, which was a randomized, multicenter, double-blind, placebo-controlled Phase III clinical trial aimed at evaluating the safety and efficacy of treating patients with Tominersen for symptomatic HD, (NCT03761849) also called GENERATION HD1. This trail recruited 791 subjects from 18 countries, who were divided evenly into three groups. One group received Tominersen every eight weeks (Q8W), while one group was given Tominersen every 16 weeks (Q16W) (receiving a placebo at alternate weeks). Another group was given a placebo only Q8W. Though the results showed that HTT protein in cerebrospinal fluid (CSF) was decreased,^[52]^ the primary outcome was not benefiting participants.^[48,53]^ Specifically, participants given Tominersen Q16W had similar scores on measures of functional ability, motor function, and cognition, at time points up to 69 weeks (about 1.5 years) of treatment compared with the placebo group. Participants given Tominersen Q8W consistently had worse scores than the other two groups. The more-frequent dosing also was associated with more severe adverse reactions. Then, the clinical trial of Tominersen was stopped in 2021. The exploratory posthoc analyses show potential benefit in younger adults with manifest HD with less disease burden at lower Tominersen exposures. GEN-EXTEND (NCT03842969) was an open-label extension study for participants who completed participation in preceding studies of tominersen (RG6042) and the dosing was also paused in 2021. New GENERATION HD1 and GEN-EXTEND data from HD treatment conference (HDTC) 2023 suggests that Tominersen at lower exposures avoids NfL increases above baseline, while still achieving CSF mHTT lowering, and has the potential for NfL lowering.^[54]^ Therefore, Roche states that it is continuing to collect and analyze data on Tominersen to look for possible paths forward. The GENERATION HD2 (NCT05686551) is a phase II, dose-finding study, which focuses on adults with prodromal or early manifest HD (stage 2 and 3 according to HD-ISS^[55]^) aged 25–50 years and with a CAP score of 400–500.^[56]^ The primary aim is to assess the safety, biomarkers, and potential clinical efficacy trends associated with lower doses of Tominersen specifically in younger adults with a lower disease burden. The GENERATION HD2 trial is testing two different, lower levels (60 mg and 100 mg given Q16W) of Tominersen without loading dose, and mathematical modeling predicts that these lower doses would be safer because they would not lead to such large increases in NfL. This study is recruiting now and eventually, there will be up to 75 sites in 15 countries.

Allele-selective mHTT suppression is made possible by population genetics studies, which have identified certain single nucleotide polymorphisms (SNPs) that are in linkage disequilibrium with the CAG expansion, referred to as HD-SNPs.^[57,58]^ HD-SNPs would provide an allele-specific mHTT gene silencing therapy for majority of HD patients worldwide.^[59,60,61]^ Researchers have identified some precise targets for allele-selective suppression of mHTT.^[60,62,63]^ Demonstrations of ASO selectivity were conducted *in vivo* using humanized HD mice, of which harboring both human mHTT and wtHTT transgenes. The preclinical therapeutic efficacy of this approach is promising, indicating the restoration of cognitive and behavioral deficits in previously impaired mice.^[62,64]^ Wave Life Sciences develops two allele-selective ASOs which target two CAG-associated SNPs, rs362307 (WVE-120101, SNP1) and rs362331 (WVE-120102, SNP2). According to the genetics of HD population, an estimated 36% to 70% of HD patients worldwide are heterozygous for at least one of the two SNPs.^[65]^ Therefore, Wave company launched two studies, namely PRECISION-HD1 (NCT03225833) and PRECISION-HD2 (NCT03225846), which aims to assess the safety and tolerability of the drugs at various dosages, ranging from 2 mg to 32 mg. The analysis shows the average reduction of CSF mHTT remained consistent across all treatment groups even in the 32-mg treatment group, indicating a lack of dose-response, and suggesting that the drug did not effectively engage the intended therapeutic target.^[48]^ In addition, NfL did not increase over time and though cUHDRS scores did not improve, they were not significantly worse than the natural history study projections suggesting that the ASO treatment was not directly associated with the exacerbation of HD progression. The analysis raises concerns about the underlying safety and tolerability of the platform. At this time, Wave does not plan to continue development of WVE-120101 and WVE-120102 but will instead focus on a different allele-selective HD ASO.^[66]^ In November 2020, Wave Life Sciences announced the development of WVE-003 (NCT05032196), an allele-selective ASO targeting a third HD-SNP that is targetable in an estimated 40% of the HD patient population.^[67]^ Building upon encouraging preclinical data demonstrating the reduction of mHTT in motor neurons and BACHD mice,^[66]^ Wave Life Sciences progressed with the development of WVE-003. The company initiated a phase I clinical trial for WVE-003 in 2021, which is called Select HD.

VO659, a CAG-expansion-specific ASO, is designed by VICO company for multiple polyglutamine disorders including spinocerebellar ataxia (SCA) type 3, type 1 and HD.^[68,69]^ By using a 2’-O-methyl phosphorothioate (2OMePS)(CUG) 7 AON that specifically targets expanded CAG stretches and does not activate RNaseH knockdown, VO659 targets the CAG repeat expansion in an allele-preferential manner, reversibly and transiently in nature, and does not change DNA code. VO659’s unique target and dual mechanism of action enable potent target engagement across multiple disease models,^[68,69]^ while they risk off-target effects through binding to another CAG repeat containing RNAs. In preclinical studies, significant and dose-dependent reductions of mHTT and improvement in motor function were observed *in vivo* in the disease mouse models of HD ^[68]^ and also *in vitro* in HD patient cell models.^[70,71]^ The Phase 1/2a clinical trial of VO659 is a multi-center, open-label basket study designed to assess the safety and tolerability of multiple ascending doses of VO659 administered intrathecally in participants with early manifest HD or mild to moderate SCA1 or SCA3 (NCT05822908). The study is expected to enroll approximately 71 participants.^[72]^ In April, 2023, Vico Therapeutics Announces first patient was dosed in phase 1/2a clinical trial of VO659 in HD.

Although evidence from mice suggests that ASO targeting outside of exon 1 is therapeutically efficacious, it is still unclear whether the alternative splice variant of HTT exon 1 [2223] would be suppressed by this ASO. ASOs exhibit their activity within the nucleus of cells by targeting pre-mRNA, enabling them to induce the degradation of transcripts before splicing occurs. This characteristic allows ASOs to potentially reduce both exon 1 splice variants and the full-length of the HTT gene. The binding sequence of Tominersen, is situated within exon 36 of HTT, positioned distally from the exon 1 transcript. Depending on the kinetics of RNA processing, the exon 1 splice variant may still be produced, potentially leading to continued pathogenic effects even in the presence of full-length HTT suppression.

ASO therapy offers several advantages including high specificity, minimal off-target effects, and the ability to target previously “undruggable” targets. ASO therapy has shown promising results in preclinical and clinical studies for various diseases, including genetic disorders, neurological diseases, and cancers. Despite the potential benefits, challenges remain in the development and delivery of ASOs, such as improving the stability, bioavailability, and specificity of the molecules and optimizing their delivery to target tissues. In non-human primate models, the direct administration of single-stranded ASOs to CSF results in widespread distribution throughout the cortical regions of the brain,^[62,73]^ which are associated with cognition. However, these ASOs are not as proficient as viral therapies in reaching subcortical structures such as the basal ganglia and limbic system, which play a role in motor function and psychiatric symptoms in HD.^[74]^ These challenges are needed to further overcome. Nevertheless, ASO therapy has emerged as a rapidly growing field with significant potential for the treatment of a wide range of diseases, including HD, due to their long half-lives, increased bioavailability, and uptake by neurons and glia.

RNA interference (RNAi) is a potentially curative therapy to employ siRNA or shRNA ^[75]^ to knock down mHTT for HD. Most HD RNAi therapies to date have been based on synthetic siRNAs or ASOs delivered naked, conjugated to cholesterol, or with lipofectamine. Unfortunately, these drugs require repeated dosing, commonly exhibit off target effects, and exert renal and hepatic toxicity.^[75]^ shRNA is a synthetic RNA molecule with a short hairpin secondary structure. Because it is delivered on a DNA plasmid rather than as double stranded RNA (*e.g*., siRNA), shRNA can be continually expressed for months or years.^[76]^ In order to optimize the delivery, the researchers from Alnylam Pharmaceuticals were working on an RNAi drug for HD using new C16 key approach. Preclinical Results on Alnylam’s Novel C16 Conjugate Technology for Delivery of siRNAs to the central nervous system (CNS) showed conjugation of 2’-O-palmityl (C16) to siRNAs along with 5’-VP enables safe, robust and durable target knockdown in the rat CNS, which could help the development for RNAi therapy in HD patients. AMT-130 is a miRNA targeting human HTT, delivered via adeno-associated viral vector serotype 5 (AAV5-miHTT), which entered the striatum through MRI-guided stereotaxic infusion and has been shown to efficiently lower normal and mutant HTT levels both *in vitro* and *in vivo* (*i.e*., rodent models).^[77,78,79]^ Two clinical trials are recruiting now to explore safety, tolerability, and efficacy in early manifest HD patients from USA and Europe (NCT04120493, NCT05243017). NCT04120493 is the first phase I/II, randomized, double-blind, multiple doses, sham control study to investigate the safety, tolerability, and efficacy of striatally-administered AMT-130 in early manifest HD started in 2019 in the United States and the second study of AMT-130 started in 2021 in Europe.

### Small molecules

The realm of small molecule interventions for HD presents an intriguing avenue of research, primarily palliative in nature.^[80,81,82,83,84,85]^ Their capacity to traverse the blood-brain barrier (BBB), extended shelf life, and ease of administration renders them particularly promising.^[86]^ This section introduces part of the collection of both preclinical and clinical studies in this domain.

Preclinical studies previously showed that intraventricular infusion of ganglioside Monosialotetrahexosylganglioside (GM1) induces phosphorylation of mHTT at specific serine amino acid residues that attenuate HTT toxicity, restoring normal motor function in already symptomatic HD mice.^[82]^ Additionally, histone deacetylases (HDACs) 4 reduction was reported to delay cytoplasmic aggregate formation, restore brain-derived neurotrophic factor (BDNF) transcript levels, and rescue neuronal and cortico-striatal synaptic function in HD mouse models.^[85]^ And, the selective Sirtuin 1 inhibitor could also alleviate HD pathology in animal models, providing the potential therapeutic treatment for HD.^[83,84]^ Nuredis develops a novel approach which is the specific transcription elongation cofactors could bind to DNA regions with long expanded CAG repeats^[87]^ to block transcription of mHTT mRNA. Spt4 could aid RNA polymerase II activity by reducing the dissociation of polymerase from the template.^[88,89,90]^ Inhibition of Spt4 and Supt4h expression was reported to successfully reduce mHTT level selectively and limit its aggregation and toxicity with no significant effect on the overall mRNA synthesis, ^[87]^ providing a novel target of disease-modifying therapies for HD. Small-molecule blockers of this pathway are currently in pre-clinical development.

Tetrabenazine and deutetrabenazine are the only small-molecule drugs approved by the Food and Drug Administration (FDA) and also in China to manage HD chorea.^[91,92,93]^ modulating dopamine by selectively inhibiting vesicular monoamine transporter type 2 (VMAT2).^[94]^ In addition to controlling the symptoms of chorea, several clinical trials (NCT04301726, NCT0471398) are also conducted to elucidate the efficacy of deutetrabenazine to control symptoms of dysphagia, functional speech and gait dynamics in HD patients, while no results have been published up to now. One another clinical trial (NCT04071639) is performed to further optimize the treatment regimen of Chinese HD patients based on different disease stages through a long follow-up period.

The strategy employed by companies such as SOM Biotech and PTC Therapeutics involves utilizing artificial intelligence and a molecular bank to identify compounds resembling Tetrabenazine or deutetrabenazine. This approach, known as drug repurposing, aims to explore new applications for existing drugs. HD patient-derived embryonic stem cells (ESCs) and induced pluripotent stem cells (iPSCs) could be used as cell models to identify small molecules that modulate HTT (or mutant HTT) protein levels for phenotypic screening.^[95]^ Here, we discuss some clinical pipeline studies of anti-HD small molecules, active or recruiting. These candidates either modulate the key drivers involved in HD pathogenesis (mHTT genesis and degradation) or downstream pathways related to excitotoxicity, proteostasis, immunomodulation, and mitochondrial dysfunction, among others, or improve the non-motor symptoms like sleep, and psychiatric problems.

Pridopidine, ^[96]^ formerly called huntexil, is a selective agonist of the sigma1 receptor (sigma1R), a molecular chaperone located in the ER in association with microconidia. Sigma1Rs are highly expressed in the CNS, and regulate calcium signaling, ion channel function, and the ER stress response, participating to increase the production of BDNF. Previous two large clinical trials (MermaiHD and HART) in patients with manifest HD indicated that pridopidine could improve motor function in voluntary movements (balance and gait), including hand movements, lowering around 3 points versus placebo in both these trials by UHDRS-TMS.^[97,98]^ Its behavioral pharmacology shows state-dependent modulation of dopamine-related psychomotor functions, paralleled by enhanced synaptic connectivity in cortico-striatal pathways. This hints at its potential to alter HD phenotypic expression and progression.^[99,100]^ Prilenia Therapeutics developed a phase 3, randomized, double-blind, placebo-controlled study, called PROOF-HD, to evaluate the efficacy and safety of pridopidine 45 mg bid in patients with early-stage HD (NCT04556656). SAGE-718, developed by Sage Therapeutics, is a derivative of the endogenous steroid 24 (S)-hydroxycholesterol. It is a positive allosteric modulator of the N-methyl-D-aspartate (NMDA) receptor, whose activity induces long-term potentiation of synapses, and thus is essential for learning and memory.^[101]^ A previous study found the 24 (S)-hydroxycholesterol in plasma decreased in HD patients, which correlated with more severe cognitive symptoms.^[102,103]^ In a multi-dosing study, with the endpoints including cognitive testing, CogState tests of working memory and executive function were improved after 11 days of SAGE-718, compared to placebo. In another phase 1 study of six patients with HD, two weeks of SAGE-718 improved working memory.^[104]^ Therefore, two placebo-controlled double-blind phase 2 studies to evaluate whether sage-718 could help cognition and functioning capacity in HD patients (NCT05107128, NCT05358821) and a phase 3, multicenter, open-label safety study to evaluate the long-term safety and tolerability of SAGE-718 in HD patients (NCT05655520) are ongoing. Dextromethorphan/quinidine (DM/Q) is a fixed-dose combination therapy that was approved by the FDA in 2010. Marketed by Avanir Pharmaceuticals as Nuedexta, it combines dextromethorphan, a noncompetitive N-methyl-D-aspartate (NMDA) receptor antagonist and sigma-1-receptor (S1R) agonist, with quinidine, which extends dextromethorphan’s plasma levels by inhibiting the CYP2D6 enzyme.^[105]^

It has been studied in many neurodegenerative diseases such as amyotrophic lateral sclerosis,^[106]^ and Alzheimer’s disease.^[107]^ Additionally, investigations have been conducted in a phase 3 clinical trial to explore its potential in treating irritability among HD patients (NCT03854019).

Branaplam is an orally available, brain penetrant, mRNA splicing modulator, which could lower total HTT (tHTT) and mHTT levels in fibroblasts, induced pluripotent stem cells (iPSC), cortical progenitors, and neurons.^[108]^ The effects of Branaplam on exon inclusion have been observed, particularly a 115 bp exon in the HTT transcript. This exon, induced by Branaplam treatment in both control and HD patients, triggers a frameshift that significantly reduces HTT RNA and protein levels. Notably, Branaplam demonstrates the ability to mitigate abnormal alternative splicing in fibroblasts and cortical neurons derived from HD patients, suggesting the potential of splicing modulators in addressing CAG repeat disorders.^[109,110]^ Novartis tested the therapy in a phase 2 clinical trial called VIBRANT-HD (NCT05111249), though dosing was suspended in August 2022 due to possible neurological side effects.

Besides, PTC 518, a mRNA splicing modulator, delivered orally, is identified to selectively and specifically modulate HTT mRNA splicing to reduce HTT protein. A phase 2 trial to evaluate the safety and pharmacodynamic effects of PTC518 is recruiting now (NCT05358717).

Valbenazine, a highly selective vesicular monoamine transporter 2 inhibitor (VMT2), is reported to significantly improve chorea and be well tolerated in the randomized, double-blind, placebo-controlled KINECT-HD trial^[111]^ (NCT04102579) including 64 patients using valbenazine and 61 patients using placebo for 12 weeks. The results showed that among the 8 subscales of the HD Health Index (HD-HI),^[112]^ the valbenazine showed greater improvements relative to the placebo at week 12. Post hoc analyses indicate that those with the largest least-square mean differences between treatment groups are abnormal movements, emotional health, hand and arm function, cognition, and social satisfaction. A statistically significant difference was found for abnormal movement at week 12 (*P* = 0.0379).^[111,113]^ Valbenazine is FDA-approved for tardive dyskinesia^[114]^ and is under investigation as a potential treatment for chorea associated with HD. Now, there is a phase 3, open-label study to evaluate the long-term safety and tolerability of valbenazine, and to provide participants continued access to valbenazine for the treatment of chorea associated with HD (NCT04400331). SOM3355 (Bevantolol hydrochloride), discovered through SOM Biotech’s proprietary artificial intelligence-based computational technology SOMAIPRO, is an alternative VMAT2 inhibitor that could be repositioned to treat chorea in HD and potentially eliminate the severe side effects of Xenazine. The first study in humans found it could reduce chorea in HD patients and have a good safety profile. So, a phase 2b, randomized, double-blind, placebo-controlled study in parallel groups assessing the efficacy and safety of two doses (300 mg/200 mg id) of SOM3355 in patients suffering from HD with chorea (NCT05475483). Melatonin has been proved to improven circadian rhythm sleep disorders, circadian rhythm sleep disorders and delayed sleep phase, which also was studied to test the efficacy. The clinical study on Melatonin for HD Gene Carriers with HD Related Sleep Disturbance-a Pilot Study (NCT04421339) has been completed and the results of the study have not yet been published. Metformin, offering cardiovascular protection and beneficial effects on obesity, musculoskeletal and reproductive diseases, cancer, and aging,^[115]^ was tested to improve the different cognitive subtests tested by UHDRS-cognitive (NCT04826692).

In prior research, it has been suggested that neurodegeneration observed in individuals with HD might potentially stem from the impaired capacity to produce cysteine.^[116]^ Disease progression was slowed in mouse models of HD fed a cysteine-rich diet. Therefore, N-Acetyl Cysteine-premanifest HD gene expansion carriers (NAC-preHD) is a phase II randomized placebo-controlled study of oral NAC among premanifest HD gene expansion carriers, with clinical and radiological outcomes at three years. (NCT05509153). Also, fenofibrate, an FDA-approved drug for high cholesterol and/or elevated triglycerides (fats) which was a kind of Peroxisome Proliferator-Activated Receptors-α (PPAR-α) agonist,^[117]^ was tested the safety and efficacy as a Treatment for HD (NCT03515213).

### Huntingtin protein degrader

Targeted protein degradation (TPD) is an innovative therapeutic approach that offers the potential to address disease-causing proteins that have traditionally been difficult to target using conventional small molecules.^[118]^ The concept of utilizing proteolysis-targeting chimeras (PROTACs) to leverage the ubiquitin-proteasome system (UPS) for protein degradation was introduced approximately 20 years ago.^[119]^ Since then, TPD has transitioned from academic research to the pharmaceutical industry, with several companies actively pursuing TPD programs in preclinical and early clinical stages of development.^[120]^ This growing interest in TPD reflects its promise as a novel therapeutic modality and highlights the potential it holds for addressing previously challenging targets and advancing the field of drug discovery and development.^[119]^ Aggregation and accumulation of polyglutamine-expanded mHTT^[2]^ presents a drug target that may be addressable by a PROTAC, as clearance of mHTT via the UPS pathway is thought to be beneficial for disease progression.^[121]^ Till now, two small-molecule TPD strategies have been reported to lower mHTT levels: a small-molecule glue approach inducing the proximity between mHTT and microtubule-associated protein 1 light chain 3a (MAP1LC3a; also known as LC3) to target it to the autophagy pathway for degradation;^[122]^ and small-molecule PROTACs that recruit mHTT aggregates to E3 ligases from the IAP family, causing the proteasomal degradation of the mutant protein.^[123]^ Arvinas company is trying to target the “soluble” huntingtin protein, the form that precedes the formation of the more solidly structured protein clumps. They are also looking for drug molecules that have a preference for mutant over normal HTT protein. *In vitro*, they showed that the PROTAC they have identified binds both the mHTT protein and the trash-labeling protein, although it was still in the exploratory stage.^[124]^

### Antibody therapy

ANX005 is a monoclonal antibody designed to inhibit C1q, the initiating molecule of the complement cascade of the innate immune system, which primarily serves as the first line of defense of the host against infection.^[125,126]^ Abnormal activation of the C1q protein is reported to play a key role in HD by causing the loss of synapses and chronic neuroinflammation, eventually damaging neurons. ANX005 aims to halt complement system activation to prevent synaptic loss in HD.^[126]^ The open-label phase 2 ANX005-HD-01 study (NCT04514367), which dosed its first patient in November 2020, evaluated the safety, tolerability, pharmacokinetics and pharmacodynamics of ANX005, delivered intravenously in 28 adults either with early HD or at-risk of the disease, has not announced the results yet. Donna Finch from Alchemab Therapeutics focused on finding naturally occurring protective antibodies in patients who are resiliently affected by mutant genes. They found ATLX-1095, the antibodies against the HTT protein are generated in resilient individuals, which binds to a fragment of the HTT protein. ATLX-1095 increases the amount of HTT cleanup being performed by microglia.^[72]^ VTX-003 designed by VectorY company, has the potential to significantly delay disease progression and maintain quality of life in patients with Huntington’s chorea. VTX-003 is a vectorized antibody consisting of a vector and a transgene carrying instructions for the cell to make therapeutic antibodies. The therapeutic antibody selectively binds mutant HTT (but not normal HTT) with the goal of removing toxic mutant HTT and restoring neuronal health. VTX-003 is currently in the discovery phase.

### Genetic modifiers

Previous studies showed that the length of the CAG repeats accounts for about 50–70% of the overall variance in the timing of onset of characteristic HD motor signs.^[9,10]^ Genome-wide association studies (GWASs) of HD have identified six DNA maintenance gene loci (including MSH3, FAN1, LIG1, MLH1, PMS1 and PMS2^[127]^) as modifiers to influence the clinical symptoms of diseases.^[15,128]^ Among these, rs557874766, an imputed single nucleotide polymorphism located within a polymorphic 9 bp tandem repeat in MSH3/DHFR was found as the variant most significantly associated with onset and progression in HD.^[129]^ Therefore, disease-modifying therapies targeting the processes of somatic repeat instability and these DNA-repair-associated genetic modifiers are potentially and promisingly developed.^[127]^

Naphthyridine-aza quinolone (NA) is reported to be a promising approach to therapeutically directly targeting the CAG repeat in HD *in vivo*.[^130,131,132^] In HD patient cells and in MSNs of HD mouse striatum, NA efficiently induces repeat contractions. Contractions are specific for the expanded allele, independently of DNA replication, require transcription across the coding CTG strand and arise by blocking repair of CAG slip-outs. More preclinical research needs to be done before the treatment in HD patients. Another strategy is to alter the expression of genetic modifiers, like MSH3. TTX-3360, an ASO delivered by intracerebroventricular (ICV) injection, targeting MSH3, a component of the DNA damage response pathway that sits upstream of mHTT to drive disease onset and progression.^[128,129]^ The preclinical data showed that MSH3 knockdown in normal mice and two mouse models of HD was safe and well-tolerated; 50% knockdown of MSH3 with TTX-3360 in HD patient-derived cell lines, and with the tool ASO targeting Msh3 in the HD mouse model, slowed or stopped expansion of mutant HTT repeats. In non-human primates, a therapeutic dose of TTX-3360 was well-tolerated with no adverse effects being observed; safety and tolerability were shown in a dose-escalation and multi-dosing non-human primates (NHP) study, suggesting a broad therapeutic index. A single dose of TTX-3360 delivered via ICV injection knocked down MSH3 by 50%–80% bilaterally in the caudate and cortex of NHPs for at least 12 weeks. Clinical Trial Application of phase 1/2a study for TTX-3360 is on the way.

## Summary and conclusions

In the years following the identification of the HTT gene in 1993,^[2]^ substantial progress has been achieved in understanding the mechanisms underlying HD.^[44]^ Progress is being made in developing current HD therapeutics and improving clinician guidance, raising global care standards.^[92,133]^ The emphasis of therapeutic interventions lies in the management of symptoms through the collaborative efforts of multidisciplinary teams, utilizing both pharmacological and non-pharmacological approaches.^[36]^ Pursuing HD-specific targets at the root cause of the disease is, therefore, the new and likely future research direction.^[134]^ The increasing interest in developing therapies that specifically target mHTT RNA and DNA reflects a current trend in HD research.^[25]^ In addition, researchers are also exploring additional therapeutic targets such as dopaminergic and glutamatergic neurotransmission, synaptic activity and aggregate inhibition. This broadened focus aims to address multiple aspects of the disease and explore potential avenues for effective treatment. RNA therapies, specifically ASOs, have exhibited promising outcomes and are currently regarded as one of the most encouraging prospects for HD therapeutics. Nonetheless, the employment of RNA targeting therapies presents several challenges. Firstly, the safety of non-selective HTT lowering remains uncertain, as it is yet to be determined whether this approach poses any risks or off-target effects in larger populations. Additionally, the singular causative role of mHTT in HD pathology is still under investigation, and it remains unclear whether permanent suppression of HTT is a requisite for therapeutic efficacy. Furthermore, given the necessity for allele-specific treatments, targeting an SNP may not suffice for addressing the diverse needs of all HD patients. Moreover, many RNA-based therapies necessitate invasive modes of administration, raising concerns regarding their tolerability and long-term effectiveness when applied to larger populations. The resolution of these challenges is likely contingent upon future research endeavors. Ongoing clinical investigations of RNAi therapies, RNA targeting small molecules, and further examination of ASO are expected to provide valuable insights into the effectiveness of RNA-based therapies for individuals with HD. DNA therapies may also enter clinical trials within the next decade, while much work still needs to be done to achieve this goal. Ensuring DNA therapies, particularly CRISPR therapies, are safe for human use will be of primary importance.^[135]^ Moreover, a few recommendations are warranted for clinical trials. To ensure consistency and comparability of results across different sites and studies, future clinical trials should incorporate methods to decrease placebo and bias effects, employ standardized and validated rating scales, and conduct rigorous statistical analyses.^[16,136]^ CAG repeats could be considered cause they could greatly influence the results of clinical trials.^[137]^ The advancement of more sophisticated assessments that can effectively capture the longitudinal cognitive changes observed in HD^[138]^ will further aid in the design of clinical trials aimed at ameliorating the cognitive decline associated with this disorder. These considerations may benefit the design of more robust pre-clinical and clinical studies.

The pursuit of future therapeutics for HD is expected to extend over several years, and the availability of treatments for HD patients may still be some time away. Nonetheless, recent advancements in the field are highly promising, indicating the possibility of entering a new era of HD therapeutics. These developments inspire optimism and suggest that significant progress is being made towards the development of effective therapies for individuals with HD.
